# Neonatal mortality in Sudan: analysis of the Sudan household survey, 2010

**DOI:** 10.1186/1471-2458-13-287

**Published:** 2013-04-01

**Authors:** Amal O Bashir, Ghada H Ibrahim, Igbal A Bashier, Ishag Adam

**Affiliations:** 1 Public Health Institute, Khartoum, Sudan; 2 School of Health Sciences, City University, London, United Kingdom; 3 Faculty of Medicine, University of Khartoum, Khartoum, Sudan

**Keywords:** Neonatal mortality, Maternal factors, Delivery complications, Sudan

## Abstract

**Background:**

Sudan is classified as having insufficient progress in achieving the Millennium Development Goal (MDG-4), where the levels of child and infant mortality are among the highest in the region and the world. This study investigated factors associated with neonatal mortality in Sudan. Neonatal death is defined as death within the first 28 days of life.

**Methods:**

This study analysed data from the Sudan Household Health Survey 2nd round, which was carried out in 2010. Total of 6,198 live-born infants delivered within the two years preceding the survey were included as the study population. Multivariate logistic regression was used to model neonatal mortality as a function of maternal health parameters, socioeconomic indicators and the sex of the child.

**Results:**

There were 189 neonatal deaths out of 6,198 live births (3.0%). In the multiple logistic regression, the factors associated with neonatal mortality were advanced maternal age (≥ 40 years; OR = 2.4; 95% CI: 1.21, 4.78, p = 0.012), poor household wealth index (OR = 1.6; 95% CI: 1.18, 2.47, p = 0.005), male child (OR = 1.8; 95% CI: 1.31, 2.42, p < 0.001), delivery of baby by Caesarean section (OR = 1.6; 95% CI: 1.78, 2.42, p = 0.013) and delivery complications (OR = 1.4; 95% CI: 1.18, 2.15, p = 0.002).

**Conclusion:**

Public health interventions which target neonatal mortality reduction should adopt a risk-factor-based approach to detect pregnancy complications early and once identified, the health system should be strengthened so that these complications can be dealt with adequately.

## Background

Globally,7.6 million children die each year from preventable causes, and of these, about 40% die in the neonatal period [1]. The vast majority of these deaths are in low- and middle-income countries such as Sub-Saharan Africa and most of them occur at home [2,3].

Even though the under-five mortality rate is declining by a rate of 2.9%, the reduction in the neonatal mortality rate of 2.1% per year lags behind the rate of reduction among older age children [1,4,5]. The high rate of the neonatal death is one of the reasons why the Millennium Development Goal (MDG-4) for reducing under-five mortality by two thirds by the year 2015 may not be achieved [6]. Unfortunately, most of the data/interventions to achieve MDG-4 were developed in rich countries where the incidence of under-five mortality is low. Data on the epidemiology of neonatal mortality were scarce in societies with higher neonatal mortalities and there is less access to cost-effective interventions to prevent them - “the inverse information and care law” [7].

Despite the fact that neonatal mortality is responsible for 40% of all under-five mortality and more than 50% of total infant mortality, it is not explicitly targeted by MDGs [8]. Addressing neonatal mortality is a major enabler to reduce child mortality and achieve the MDG-4 target [3].

Understanding the factors associated with neonatal mortality is important so that public health intervention efforts to prevent neonatal mortality can be properly focused, based on the evidence [3]. In addition, the availability of valid epidemiological information at the country level will be an important determinant of success in meeting and in measuring progress toward the MDGs for child survival [1].

The causes of neonatal mortality vary between (and within) countries [2,9]. There is a scarcity of information about the direct causes of neonatal deaths in low-income communities; population-based information in these settings is largely dependent on verbal reports of autopsies of variable quality. Global estimates are only possible through statistical modelling [2].

Sudan is classified as having made insufficient progress to achieve MDG-4 [1]. The current infant mortality rate is 60 per 1,000 live births and the under-five mortality rate is 82 deaths per 1,000 live births. The neonatal mortality rate is also high ranging from 34 to 47 per 1,000 births [10].

This aim of this study was to investigate the factors associated with neonatal mortality in Sudan. Such data are of paramount importance, providing the evidence necessary to guide future interventions to reduce the infant death rate.

## Methods

### Data sources

**Figure 1:**
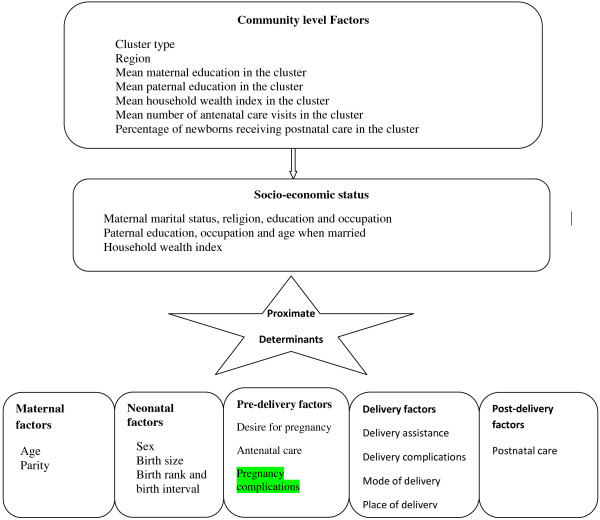
Conceptual framework for factors influencing neonatal mortality.

Data collected by the Sudan Household Health Survey 2nd round (SHHS2, 2010), which was carried out in 2010, were used in this study [10]. This survey collected information on children and women, measuring key indicators that allow the country to monitor progress towards MDGs and other internationally agreed upon commitments. Although SHHS2, 2010 covered both Northern and Southern Sudan, only the data covering the 15 Northern States was used in this study (Figure 1). This was due to the Southern States’ data being unavailable. In each state, 25 households from each of 40 clusters (villages) were sampled. A two-stage cluster sampling design was employed to choose the samples in each state. Questionnaires were based on the Standard Multiple Indicator Cluster Survey, fourth round (MICS4) and the Pan Arab Project for Family health (PAPFAM) questionnaires [11], which were adapted and amended to suit the country.

For this study, data were obtained from the Women’s Questionnaire (for women aged 15–49 years), which included information on demographic characteristics, and then details of births within the 2 years preceding the survey - child mortality, history of antenatal care, delivery assistance, and mode and place of delivery for births.

### Conceptual framework

**Figure 2:**
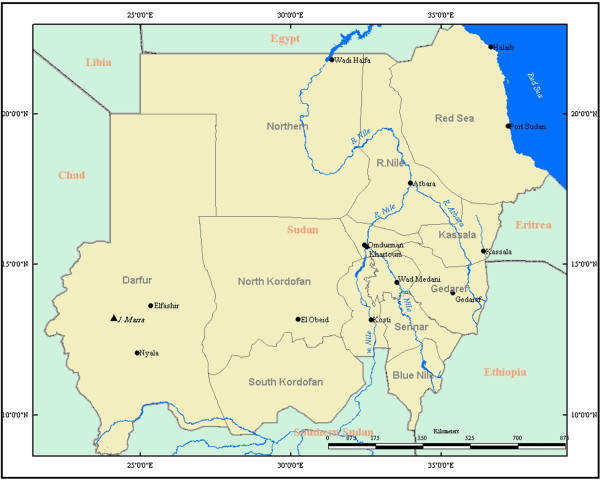
Map of Sudan.

A previously designed conceptual framework for the study of child survival in developing countries [3,12] was adopted and slightly modified based on the available data in the SHHS2, 2010, for identifying independent variables (Figure 1). Some factors were not available from the SHHS2, 2010, so they were excluded from the analysis (Figure 2) (e.g. community-level factors and post-delivery factors).

### Study population

Live-born infants delivered within the 2 years preceding the survey (i.e. February 2008 onwards) were included as the study population (n=6,198).

### Description of variables

Table 1 shows a list of all the variables used in this study along with their definitions and categorisations. Neonatal mortality is defined as the death of a child within the first 28 days of their life. It was employed as a binary outcome variable in the analysis.

**Table 1:** Definition and categories of variables used in the analysis into neonatal mortality rates in Sudan

**Variable**	**Definition and categories**
Child sex	Male /female
Maternal age in years	Original variable present the mother’s age as continues variable. However, in the analysis mother’s age was entered as a categorical variable in which categorized into four age groups <20; 20–29; 30–39; ≥40 yrs.
Maternal education	Variable was constructed from two variables; 1st is ever attended school. Women who answered “yes” were asked the second about the highest level she completed. Women who reported that they only completed the preschool were considered as illiterate and added to women who reported in the first question that they have never attended school. Women who attended informal education such as “ illiterate eradication classes” are considered in the primary level and then they were added to the women who reported that they attended primary or intermediate levels (the basic education) Women who reported that they attended secondary or university levels were grouped together to be the third group. Categories were; illiterate, intermediate or less (≤8 years) and secondary or above (>8 years)
Area of residence	Rural/ urban
Household head education	Because of the very high percentage of missing in the education level variable, only ever vs. never had some education variable has been entered to the analysis as an indicator that lead us to know the role of education of head of household in any health decision taken. Illiterate / some education.
Household wealth index	The original variables has 5 categories ranged from poorest (the 1st quintile) to richest (the 5th quintile). Because of the very small numbers of neonatal deaths in the second and forth quintiles, the 2nd and the 1st quintiles were grouped together as well as the 4th and the 5th quintiles were grouped together. Therefore, the wealth index that used in the analysis contains three categories: poor, middle, and rich.
Parity	The original variable was continuous it was group in to three categories; primiparae, 2–4 children and multiparae ≥ 5.
Antenatal care utilization and delivery assistance	Were constructed from a multiple 9 alternative health providers. Women who answered: Traditional birth attendant or Community health worker, no one was coded as non / traditional health providers (THP) users. whereas women who answered doctor or nurse or village midwife or health visitor or medical assistant were coded professional health providers (PHP) users. However, if women mentioned that they visited both health provider belongs to THP group and one belongs to PHP group, they were considered as PHP users.
Ever had tetanus toxoid vaccination	Yes / No
Mode of delivery	The original variable has three categories: vaginal, Caesarean section, forceps extractor. Because of the very few cases in the forceps extractor, they were added to vaginal cases. Thus the variable that use in the analysis includes vaginal and Caesarean section
Place of delivery	Home/ health facility
Pregnancy complication and delivery complications	Women were asked about their personal experience in their last pregnancy and last delivery. The variables were constructed from the responses of the: excessive vaginal bleeding, high blood pressure, convulsions, high fever, foul smelling vaginal discharge, and Jaundice in which women who reported yes in at least one of these complications during pregnancy period / delivery were coded 1 “yes” whereas women who reported that did not experienced any of these complications were coded 0 “No”.

### Demographic and socio, economic variables

These incorporated sex of child; maternal age in years (<20, 20–29, 30–39, ≥40 yrs); maternal education level (illiterate, intermediate or less (≤8 years) and secondary or above (>8 years); head of household education (illiterate, have some education), area of residence (rural, urban); household wealth index (high, middle, low).

### Maternal health variables

The included variables were; parity (primiparae, 2 to 4 children, multiparae≥5); antenatal care utilization (ANC) (non/receiving traditional health care, and receiving professional health care); delivery assistance was treated in the same way as the ANC variable; mode of delivery (vaginal, Caesarean section); place of delivery (home, health facility deliveries); ever had tetanus toxoid vaccination (yes, no); ever experienced complication during last pregnancy (yes, no); ever experienced complication during last delivery (yes, no).

### Ethics

Ethical permission for the study was obtained from the Federal Ministry of Health Ethical Committee, Khartoum, Sudan.

### Statistical analyses

First, descriptive analysis using frequency tabulation was conducted. Crude odds ratios and 95% confidence intervals were calculated to assess the rough effect of each explanatory variable on the neonatal mortality rate without adjusting for other variables. Variables found to be significantly associated with neonatal mortality on at least one category (defined as a p value < 0.05) were entered into a multivariate logistic regression model to evaluate neonatal mortality as a function of demographic, socioeconomic and maternal health indicators. Pregnancy complications were removed from the final model because it was highly correlated to delivery complications. Similarly, parity was removed from the final model because it was highly correlated to maternal age. The final model included maternal age, household wealth index, sex of child, mode of delivery and delivery complications.

The statistical analyses were performed using SPSS® version 18 for Windows (SPSS Inc., Chicago, IL, USA).

## Results

In total, 21,942 women were interviewed, and of the 6,198 live-born children who were included in the analysis, 189 (3.0%) had died during the neonatal period. Characteristics of the study population are presented in Tables 2 and 3. Approximately 51% of the children were male, 52% of the children were born to a mother in the 20–29 years age group, 73% were born to mothers who lived in rural areas, 45% were born to mothers who were illiterate 49% of the heads of the household were illiterate and 46% of the children were born into poor families (lowest wealth index).

**Table 2:** Details of births and neonatal deaths recorded in the Sudan household health survey 2nd round, 2010 (N = 6,198)

**Characteristics**	**Number of children**	**%**	**Neonatal mortality rate (per 1000 live births )**
**Child sex**			
Male	3152	50.9	38
Female	3046	49.1	22
**Maternal age in years**			
<20	458	7.4	26
20 – 29	3217	51.9	30
30 – 39	2075	33.5	26
≥40	448	7.2	60
**Maternal education**			
Illiterate	2803	45.2	34
Intermediate or less(≤8 years)	2366	38.2	28
Secondary or above (> 8 years)	1029	16.6	27
**Area of residence**			
Urban	1674	27.0	31
Rural	4524	73.0	31
**Household head education**			
Illiterate	3020	48.7	32
Have some education	3140	50.7	28
missing	38	0.6	
**Household wealth index**			
Lowest	2860	46.1	37
Middle	1366	22.0	26
Highest	1972	31.8	24

**Table 3:** Maternal characteristics of the babies recorded in the Sudan household health survey 2nd round, 2010 (N = 6198)

**Characteristics**	**Number of children**	**%**	**Neonatal mortality rate (per 1000 live births )**
**Parity**			
primiparae	1129	18.2	53
2-4 children	2988	48.2	24
Multiparae ≥5	2080	33.6	27
missing	1	0.0	
**Antenatal care utilization**			
Non/ traditional care	1605	25.9	35
Professional health care	4593	74.1	29
**Ever has tetanus toxoid vaccination**			
Yes	4356	70.3	31
No	1842	29.7	30
**Delivery assistance**			
Non/ traditional care	1634	26.4	35
Professional health care	4477	72.2	29
missing	87	1.4	
**Mode of delivery**			
Vaginal	5686	91.7	29
Caesarean section	406	6.6	47
missing	106	1.7	30
**Place of delivery**			
Home	4834	78.0	30
Health facility	1263	20.4	31
missing	101	1.6	
**Pregnancy complications**			
Yes	2835	45.7	39
No	3363	54.3	24
**Delivery complications**			
Yes	2250	36.3	37
No	3948	63.7	27

Eighteen per cent of the mothers were primiparae, 74% of the mothers received professional antenatal care, 70% had received at least one dose of anti-tetanus vaccine, 92% of the deliveries were vaginal, 78% delivered at home, 72% had professional assistance during delivery, 46% experienced complications during pregnancy and 36% experienced complications during delivery.

### Crude odds ratios

Factors significantly associated with neonatal mortality were: older maternal age (≥ 40 years), OR 2.3 compared with age < 20 years, (95% CI: 1.16, 4.55, p = 0.017), primiparity OR 2.3, compared with having 2–4 children, (95% CI: 1.61, 3.23, p < 0.001), lowest household wealth index OR 1.6 compared with highest wealth index, (95% CI: 1.11, 2.21, p = 0.012), child of male sex OR 1.6 (95% CI: 1.30, 2.38, p = 0.017). Pregnancy and delivery complications had a significant association with neonatal mortality (pregnancy complications OR 1.7, (95% CI: 1.24, 2.23, p = 0.001; delivery complications OR 1.4 (95% CI: 1.06, 1.90, p = 0.018). Babies born by Caesarean section were more likely to die before age 28 days than babies born by vaginal delivery OR 1.6 (95% CI: 0.98, 2.60, p=0.06).

Maternal education, place of residence and the level of education of the head of the household were not significantly associated with neonatal mortality. In addition no significant associations were found with the other maternal health factors: use of antenatal care, tetanus vaccination, assisted delivery or place of delivery, Table 4.

**Table 4:** Crude and adjusted odds ratios for factors associated with neonatal mortality

**Factor**	**Crude OR (95%CI)**	**P value**	**Adjusted OR (95% CI)**	**P value**
**Maternal age in years** (n= 6301) (R^2^=0.023)**				
<20	1		1	
20 – 29	1.1 (0.61, 2.00)	0.752	1.1 (0.60, 2.00)	0.758
30 – 39	0.9 (0.50, 1.75)	0.841	0.9 (0.49, 1.72)	0.777
≥40	2.3 (1.16, 4.55)	0.017	2.4 (1.21, 4.78)	0.012
**Maternal education** (n= 6301) (R^2^= − 0.017)**				
Secondary or above (>8 years)	1			
Intermediate or less (≤8 years)	1.0 (0.65, 1.60)	0.919		
Illiterate	1.2 (0.82, 1.92)	0.302		
Parity* (n= 6301) (R^2^= 0.013)**				
2-4 children	1			
primiparae	2.3 (1.61,3.23)	<0.00		
Multiparae ≥5	1.1 (0.10 , 1.61)	0.493		
**Household wealth index** (n= 6301) (R^2^= − 0.034)**				
Highest	1		1	
Middle	1.1 (1.11 , 2.21)	0.834	1.1 (0.69 , 1.72)	0.710
Lowest	1.6 (1.11 , 2.21)	0.012	1.6 (1.18 , 2.47)	0.005
**Place of Residence** (n= 6301) (R^2^= − 0.001)**				
Rural	1			
Urban	1.0 (0.74 , 1.41)	0.918		
**Child sex** (n= 6301) (R^2^= − 0.047)**				
Female	1		1	< 0.001
Male	1.6 ( 1.30 , 2.38)	0.017	1.8 (1.31 , 2.42)	
**Household head education** (n= 6263) (R^2^= − 0.007)**				
Have some education	1	0.344		
Illiterate	1.2 (0.86 , 1.55)			
**Antenatal care utilization** (n= 6301) (R^2^= − 0.016)**				
Professional health care	1			
Non/ traditional care	1.2 (0.89 , 1.68)	0.211		
**Ever has tetanus toxoid vaccination** (n= 6301) (R^2^= 0.001)**				
Yes	1			
No	1.0 ( 0.72 , 1.36)	0.948		
**Delivery assistance** (n= 6217) (R^2^= − 0.016)**				
Yes	1			
No	1.0 ( 0.72 , 1.36)	0.948		
**Mode of delivery** (n= 6301) (R^2^= 0.024)**				
Vaginal	1		1	
Caesarean section	1.6 (0.98 , 2.60)	0.060	1.6 (1.78 , 2.42)	0.013
**Place of delivery** (n= 6206) (R^2^=0.002)**				
Home	1			
Health facility	1.0 (0.72 , 1.46)	0.897		
Pregnancy complications* (n= 6301) (R^2^=0.044)**				
No	1			
Yes	1.7 (1.24 , 2.23)	0.001		
**Delivery complications** (n= 6301) (R^2^=0.030)**				
No	1		1	
Yes	1.4 (1.06 , 1.90)	0.018	1.4 (1.18, 2.15)	0.002

*Excluded from the model because of co linearity. ** for the crude analysis.

Nagelkerke R^2^ for the final model = 0.060.

### Adjusted odds ratios

In the multivariate analysis, the risk of neonatal mortality increased significantly with advanced maternal age OR 2.4 (95% CI: 1.21, 4.78, p = 0.012) for women in age group ≥ 40 years, compared with women < 20 years. Low household wealth was associated with increased odds of neonatal mortality compared with high household wealth (OR 1.6; 95% CI: 1.18, 2.47, p = 0.005). Male babies have 1.8 times higher odds for neonatal mortality than female babies (95% CI: 1.31, 2.42, p < 0.001). Babies born by Caesarean section have 1.6 higher odds of neonatal mortality than those born by vaginal delivery (95% CI: 1.78, 2.42, p = 0.013). Delivery complications were associated with 1.4 higher odds for neonatal mortality compared with those delivered free of complications (95% CI: 1.18, 2.15, p = 0.002) Table 4.

## Discussion

Analysis of data from the SHHS2, 2010 has revealed that neonatal mortality is associated with male babies, advanced maternal age, family poverty, delivery of baby by Caesarean section and delivery complications. Advanced maternal age has been associated with preterm delivery and antepartum complications in another study [13] and further studies have found that delivery complications were determinant of poor perinatal outcomes [14,15]. Obstetric complications, particularly in labour, have been reported as risk factors for stillbirths and early neonatal deaths [6,16] and poverty and delivery complications have also been identified as risk factors for neonatal death [7].

In the current study, maternal education was not associated with neonatal mortality, which is in contrast with another study which has found maternal education to be an important determinant of maternal and perinatal outcomes [17]. This may be due to mothers with higher levels of education using more antenatal care [18], however, in Sudan, this may not be the case, as women have no authority in the decision to seek medical care, even if they are educated.

The current study showed that primiparity was associated with neonatal mortality. In contrast, multiparity (≥ 5) has been reported as a risk factor for neonatal mortality [3]. Another result from the current study was the high neonatal mortality risk associated with babies born by Caesarean section. Most Caesarean section may be emergency sections following prolonged attempted vaginal deliveries.

Unexpected findings from the study were the lack of association between neonatal mortality and the use of antenatal health care services, whether the delivery was assisted by a health care professional or not and whether the birth was at home or in a hospital. A Previous study reported that lack of antenatal care was associated with a higher risk of neonatal mortality [19], and a lack of antenatal care was reported as a determinant of poor perinatal outcomes in central Sudan [17]. Further studies [7,20] have revealed that the majority of neonatal mortality occurs at home with unskilled birth attendance. These findings may reflect the poor quality service provided by nurses and midwives who are still training as birth assistants [21]. Another explanation for this result may be that in Sudan most deliveries start at home and are referred to health facilities as a result of complications e.g. prolonged labour, intra-partum haemorrhage. Thus, hospital deliveries are more complicated and more likely to have poor outcomes. Further study of the quality of services provided and the referral system for hospitals is needed.

This study also found no association between neonatal mortality and the mother ever receiving a tetanus vaccination. The survey question asked the mothers about ever receiving the vaccination, so was not checking for compliance on a course of vaccination, or recent vaccination and a positive answer was not indicative of full protection against tetanus.

In general, the study identified some risk factors for neonatal mortality that are not easily or readily modifiable. These risk factors may underlie the persistently high neonatal mortality rate seen in Sudan, despite the efforts made to reduce it. Identification of these causes of neonatal death is essential so that evidence-based approaches for reducing neonatal mortality in Sudan can be developed.

The main strength of this study is that SHHS2, 2010 was a nationally representative survey, using standardised methods that achieved high individual (91.4%) and household (99%) response rates.

One limitation of this study is that in SHHS2, 2010, maternal health indicators were collected from women on their most recent baby delivered within the 2 years preceding the survey. As a result, neonatal mortalities for children born prior to February 2008 were not included in the analysis. In addition, 189 neonatal deaths is a relatively small sample from which to draw definitive conclusions - a larger sample would have provided more power to detect associations between neonatal death and potential risk factors.

## Conclusion

The analysis of the SHHS2, 2010 data revealed that neonatal mortality is associated with poverty, advanced maternal age, having a male child, having a Caesarean section and having complications during the pregnancy. Public health interventions targeting the reduction of neonatal mortality should adopt a risk-factor-based approach to detect pregnancy complications early and to strengthen the health system to deal with problems adequately.

## Competing interests

The authors have no competing interests to declare.

## Authors’ contributions

AOB and IA designed the study. GHI and GAB participated in the survey and statistical analyses. All the authors helped draft and approved the final version of the paper.

## Pre-publication history

The pre-publication history for this paper can be accessed here:

http://www.biomedcentral.com/1471-2458/13/287/prepub
